# *Formononetin* Mediates α7nAChR to Inhibit Macrophage Polarization and Ameliorate Atherosclerotic Plaque

**DOI:** 10.2174/0115680266382024250730194614

**Published:** 2025-08-12

**Authors:** Li Du, Shirong Li, Qiansong He, Min Zhang, Wenxiu Wang

**Affiliations:** 1 Department of Pediatric, The Second Affiliated Hospital of Guizhou University of Traditional Chinese Medicine, Guiyang 550001, Guizhou, China;; 2 Department of Neurology, Guizhou Provincial People's Hospital, Guiyang 550001, China;; 3 First Clinical Medical College, The First Clinical Medical College, Guizhou University of Traditional Chinese Medicine, Guiyang 550001, Guizhou, China

**Keywords:** Formononetin, α7nAChR, M1, M2, JAK/STAT, Atherosclerotic plaque

## Abstract

**Objectives:**

To explore the molecular mechanism of α7 nicotinic acetylcholine receptor (α7nAChR) mediated by *Formononetin* (*FMN* ) in inhibiting macrophage inflammatory polarization and stabilizing atherosclerotic plaque.

**Methods:**

SiRNA α7nAChR was transfected into THP-1-induced M0 cells and treated with *FMN* . Oil Red O staining was used to evaluate macrophage lipid deposition. RT-qPCR was used to detect α7nAChR, COX-2, IL-1β, IL-6, HO-1, and SHIP1 expression in M1 and M2 macrophages. Western blot was used to detect α7nAChR, iNOS, CD206, CD68, p-JAK2, and p-STAT3 protein expression in M1 and M2 macrophages.

**Results:**

Compared with the control group, *FMN* -mediated α7nAChR reduced lipid deposition in M1 and M2 macrophages. RT-qPCR results showed that *FMN* intervention significantly downregulated COX-2 and IL-1β expression in M1 (*P* < 0.05). α7nAChR expression significantly reduced COX-2, IL-6, and IL-1β expression in M2 (*P* < 0.05) and significantly increased HO-1 and SHIP1 expression (*P* < 0.05). *FMN* -mediated α7nAChR significantly decreased the expression of iNOS, CD68, p-JAK2, and p-STAT3 in M1 and M2 macrophages and significantly increased the expression of CD206 protein by Western blot (*P* < 0.05).

**Discussion:**

This study, for the first time, elucidated the mechanism of FMN regulating macrophage polarization through the α7nAChR/JAK2/STAT3 axis, providing new experimental evidence for the role of the cholinergic anti-inflammatory pathway in cardiovascular diseases. However, there are some limitations, such as the limited applicability of the THP-1 cell line, the need to strengthen the dose correlation study, the bioavailability and solubility limiting clinical translation, and the lack of human toxicological data.

**Conclusion:**

*FMN* effectively modulates macrophage polarization through inhibition of the JAK/STAT signaling pathway while promoting α7nAChR expression.

## 1 INTRODUCTION

Atherosclerosis (AS) is a chronic disease in which lipids (mainly low-density lipoproteins) in the blood are deposited under the inner lining of the arteries due to damage, resulting in atherosclerotic plaques that thicken, harden, and lose elasticity in the artery walls [1]. AS is the leading cause of cardiovascular events such as heart attacks and strokes, killing 17.9 million people worldwide each year [2]. The progression of atherosclerotic lesions involves the buildup of lipids in the arterial intima, leading to the formation of foam cells, local inflammatory reactions, and the migration and proliferation of various cell types, including macrophages [3]. M1-type macrophages become activated in response to stimuli such as bacterial lipopolysaccharide (LPS) or interferon (IFN)-γ, resulting in elevated levels of pro-inflammatory cytokines (*e.g*., TNF-α, IL-6, and IL-12) and nitric oxide synthase (iNOS) [4, 5]. Conversely, M2 macrophages are activated by stimuli like IL-4 or IL-13, leading to increased levels of anti-inflammatory cytokines (*e.g*., IL-10 and TGF-β) and arginase-1 (Arg-1) [5]. Within atherosclerotic plaques, M1-type macrophages contribute to plaque instability and rupture by releasing pro-inflammatory cytokines and matrix metalloproteinases (MMPs), while M2 macrophages assist in reducing inflammation and enhancing plaque stability through the secretion of anti-inflammatory cytokines that support tissue organization and reconstruction [6]. Maintaining a balance between M1 and M2 macrophages is critical for regulating the inflammatory response, as cytokines play a significant role in arterial wall inflammation and can influence plaque stability [6, 7]. Therefore, modulating the inflammatory response is essential in the management of atherosclerosis.

The alpha-7 nicotinic acetylcholine receptor (α7nAChR) is a subtype of acetylcholine receptor that is widely distributed throughout the central nervous system and peripheral tissues, including macrophages and the arterial wall [8]. Within the pathophysiological microenvironment of atherosclerotic plaques, this receptor serves as a key regulator of macrophage-mediated inflammatory cascades. Pharmacological activation of macrophage α7nAChR initiates a potent anti-inflammatory signaling cascade through JAK2/STAT3 pathways, effectively downregulating pro-inflammatory cytokine secretion (*e.g*., IL-6, TNF-α) while upregulating anti-inflammatory mediators like IL-10 [8, 9]. This receptor-mediated immunomodulation establishes an anti-inflammatory milieu within developing plaques, potentially decelerating atherogenesis through multiple mechanisms [9]. Notably, α7nAChR activation exerts bidirectional modulation of macrophage polarization states: it suppresses M1 pro-inflammatory differentiation through NF-κB pathway inhibition while promoting M2 anti-inflammatory polarization *via* STAT6 activation [8-10]. This phenotypic switching enhances plaque stability through extracellular matrix remodeling and fibrous cap reinforcement by M2-polarized macrophages, substantially reducing rupture susceptibility [11, 12].

The central role of inflammatory mechanisms in the pathogenesis of atherosclerosis has been extensively reported [12]. Although conventional therapies such as statins and aspirin demonstrate clinically relevant anti-inflammatory effects, their therapeutic application is constrained by potential adverse reactions including gastrointestinal disturbances, hepatorenal toxicity, and hypersensitivity responses [12-14]. In contrast, Chinese herbal medicine has gained recognition as a promising therapeutic strategy for atherosclerotic management, characterized by its multi-target pharmacological actions encompassing lipid modulation, oxidative stress reduction, and blood pressure regulation, while maintaining a favorable safety profile [15, 16]. Contemporary pharmacological research has validated the efficacy of traditional Chinese medicine in atherosclerosis prevention and treatment, particularly highlighting its advantages in minimizing adverse effects while achieving therapeutic outcomes through synergistic multi-component interactions [17, 18].

*Formononetin (FMN)* is a phytoestrogen isoflavone compound present in the diet, which is the main active ingredient of the legume *Red Clover* and one of the active ingredients of commonly used Chinese herbal medicines such as *Angelica sinensis*, *Astragalus membranaceus*, *Radix puerariae*, and *Caulis spatholobi* [19]. After oral administration, *FMN* can be hydrolyzed along the entire gastrointestinal tract and absorbed in the proximal part of the intestine by passive diffusion. After absorption, *FMN* is rapidly O-demethylated to daidzein, which is then converted by the corresponding transferase to β-glucuronate or sulfate conjugated at one or both (4' or 7') positions of the isoflavone ring, and daidzein is then converted by the corresponding transferase to β-glucuronate or sulfate. It has good safety [20]. *FMN* is mainly isolated and obtained from Astragalus root in traditional Chinese medicine and has been used for a long time to treat diabetes, nervous system diseases, and cardiovascular diseases [21, 22]. Its pleiotropic pharmacological properties, including potent anti-inflammatory activity, free radical scavenging capacity, and platelet aggregation inhibition, position it as a promising candidate for managing diverse pathological conditions ranging from oncological and neurodegenerative disorders to fibrotic diseases and cardiovascular pathologies [19].

Research has demonstrated that *FMN* can suppress the production of proinflammatory factors such as tumor necrosis factor-alpha (TNF-α), interleukin 6 (IL-6), and interleukin 1 beta (IL-1β), reduce macrophage inflammatory responses, and alleviate plaque inflammation [23]. Moreover, *FMN* has been shown to inhibit the JAK/STAT signaling pathways associated with autoimmune and inflammatory diseases [24, 25]. Notably, Relevant studies have reported that *FMN* may exert anti-tumor effects by acting on CXCL12, ESR1, and IGF1, and may have potential synergistic effects with immune checkpoint inhibitors [26]. These findings suggest that *FMN* has a certain regulatory function on the immune system and inflammation. Our previous study found that *FMN* could reduce inflammation and atherosclerosis by regulating macrophage polarization, inhibiting the JAK/STAT signaling pathway, and promoting α7nAChR expression in ApoE-/-mice [27]. However, the precise molecular mechanisms underlying *FMN*' stabilization of atherosclerotic plaques through α7nAChR-mediated inhibition of macrophage inflammation remain unclear. Therefore, this study aims to investigate this mechanism comprehensively by utilizing siRNA technology to disrupt the α7nAChR gene in macrophages and evaluate lipid levels, inflammatory markers, and molecular pathways following *FMN* treatment. The study seeks to elucidate the anti-atherosclerotic properties of *FMN* and its potential therapeutic implications in atherosclerosis management, emphasizing the medicinal potential of natural compounds and providing a theoretical basis for the use of *FMN* in atherosclerosis prevention and treatment.

## 2 MATERIALS AND METHODS

### 2.1 Reagents

*Formononetin* (HY-N0183, MCE), Trizol Reagent (CW0580S, CWBIO), ultra-pure RNA extraction kit (CW0581M, CWBIO), CCK-8 Cell Proliferation Test Kit (KGA317, KeyGEN), ECL ultra-sensitive luminescent liquid (Thermo Fisher), BCA protein quantification kit (Elabscience), PVDF membrane (IPVH00010, Millipore), Rabbit Anti iNOS (18985-1-AP, Proteintech), Rabbit Anti CD206 (DF4149, Affinity), Rabbit Anti CD68 (DF7518, Affinity), Rabbit Anti α7nAchR (bs-1049R, Bioss), Rabbit Anti p-JAK2 (AF3022, Affinity), Rabbit Anti p-STAT3 (AF3293, Affinity), OCT embedding agent (4583, SAKURA), saturated oil Red O staining solution (G1260, Solarbio). TNF-α Elisa kit (ml077385, Meimian), IL-1β Elisa kit (ml106409, Meimian), IL-10 Elisa kit (ml064299, Meimian), IL-13 Elisa kit (ml063719, Meimian).

### 2.2 Cellular Induction and Drug Intervention

The THP-1 monocytic cell line (BNCC358410, North Carolina Biology) was maintained in complete RPMI-1640 medium supplemented with 10% fetal bovine serum (FBS) and 0.05 mM β-mercaptoethanol (HY-Y0326, MCE). To differentiate THP-1 cells into M0 macrophages, cells were treated with 320 nmol/L phorbol 12-myristate 13-acetate (PMA, HY-18739, MCE) for 30 hours. M1 macrophage polarization was achieved by stimulating M0 macrophages with 20 ng/mL interferon-γ (IFN-γ, HY-P7025A, MCE) and 100 ng/mL lipopolysaccharide (LPS, HY-D1056, MCE) for 48 hours, while M2 polarization was induced using 20 ng/mL interleukin-4 (IL-4, HY-P70445, MCE) and interleukin-13 (IL-13, HY-P7033, MCE) for the same duration. The experimental design comprised three treatment groups: M1/M2 control group, M1/M2 + scrambled siRNA (si-NC) + *Formononetin* group, and M1/M2 + α7nAChR siRNA (si-α7nAchR: si-219#1, si-289#2, and si-391#3) + *Formononetin* group. Prior to polarization, M0 macrophages were transfected with either 40 nM of si-nc or si-α7nAchR using LipoRNAi transfection reagent (Beyotime, C0535). The transfection mixture was prepared by directly combining serum-free medium, siRNA, and LipoRNAi transfection reagent, followed by incubation at room temperature for 20 minutes. The mixture was then added directly to the cell culture vessel. After six hours of transfection, the culture medium was replaced. Subsequently, the cells were induced to polarize into either M1 or M2 phenotypes. Following polarization, the cells were treated with *Formononetin* for 48 hours before subsequent analysis.

### 2.3 Immunofluorescence

After fixation with 4% paraformaldehyde, cell slides were permeabilized with 0.1% Triton X-100. This was followed by blocking with 5% BSA for 1 h, followed by the addition of primary antibodies and incubation at 4 °C overnight. After the addition of fluorescent secondary antibody for 1 hour at room temperature in the dark, the cells were counterstained with DAPI in the dark and observed under a fluorescence microscope after sealing.

### 2.4 ELISA Assay

The cell supernatant was centrifuged at 1000×g for 10 min to remove particles and polymers. The procedure was performed according to the instructions of the kit. Specimens were diluted 1:1 with specimen diluent and added to 50 µL in the reaction wells. Add 50 µL of the diluted standard to the reaction well, and add 50 µL of the tested sample to the reaction well. Fifty µl of the biotin-labeled antibody was added immediately. The membrane plates were covered, mixed with gentle shaking, and incubated at 37 °C for 1 hour. Subsequently, washing was performed. Avidin-HRP was added and incubated at 37 °C for 30 min before washing. 50 µL of each substrate A and B were added to each well, mixed with gentle shaking, and incubated at 37 °C in the dark for 10 minutes before swiftly adding 50 µL of termination solution. The OD values of each well were determined at a wavelength of 450 nm.

### 2.5 Cell Viability Assay

Following the treatment of cells with varying concentrations of *FMN* (0µM, 2µM, 5µM, 7.5µM, and 10µM) for a duration of 48 hours, a 10% CCK-8 solution was introduced to each well and subsequently incubated in the dark for 2 hours. The absorbance at 450 nm was then quantified using a microplate spectrophotometer. Cell viability was calculated as the following formula (Eq.1):

Cell viability = [(A_450, blank_) / (A_450, blank_)] × 100% (1)

### 2.6 Oil Red O Staining

After fixation, oil red O staining, and isopropyl alcohol differentiation, the cells were re-stained with hematoxylin dye, and the excess dye was removed. The lipid deposition in the cells was observed under the microscope.

### 2.7 RT-qPCR

Total RNA was extracted, and the first template chain was reversed according to the kit instructions. Subsequently, fluorescence quantitative PCR was performed. GAPDH served as the internal reference, and the relative gene expression levels were determined using the 2^^-ΔΔCt^ method. The sequences of the primers utilized are presented in Table **1**.

### 2.8 Western Blotting

Total cellular protein was collected, and protein concentration was quantified by BCA assay. Thirty µg of protein was electrophoresed by SDS-PAGE for 1.5 h, followed by membrane transfer at a constant current of 300 mA for 1 h. PVDF membranes blocked with skim milk powder were combined with primary antibodies and incubated overnight at 4 °C. The next day, the cells were incubated with secondary antibodies for 2 h at room temperature. Finally, chemiluminescence reagents were combined with the ultrasensitive chemiluminescence imaging system for visualization.

### 2.9 Statistical Analysis

All experiments were statistically analyzed and plotted using GraphPad Prism 8.0.1 software with three repetitions. Quantitative results are expressed as X±S. One-way analysis of variance was used for quantitative comparison among multiple groups, and the significance level was set at α=0.05. *P* < 0.05 was considered statistically significant.

## 3 RESULTS

### 3.1 The siRNA Technique Interferes with the Expression of α7nAChR in M1 and M2 Macrophages

THP-1-derived monocytes were differentiated into M0 macrophages. The secretion levels of M1 polarization-related cytokines, TNF-α and IL-1β, were significantly elevated in the M1-induced group compared to the normal control group (*P* < 0.05), confirming successful M1 polarization of macrophages. Similarly, the M2-induced group exhibited markedly higher levels of M2-associated cytokines, IL-10 and IL-13, relative to the control group (*P* < 0.05) (Fig. **1**), demonstrating effective M2 polarization. Furthermore, immunofluorescence analysis was performed to validate the expression of specific polarization markers, providing additional confirmation of macrophage phenotypic differentiation. Immunofluorescence analysis confirmed the successful induction of M0 macrophages, as evidenced by the expression of the macrophage-specific marker CD68 (Fig. **2A**). The presence of iNOS confirmed the polarization of M0 macrophages toward the pro-inflammatory M1 phenotype (Fig. **2B**), while CD206 expression verified their differentiation into anti-inflammatory M2 macrophages (Fig. **2C**). Compared with the control group, the mRNA and protein levels of α7nAChR in each group were significantly decreased after siRNA interference (*P* < 0.05) (Fig. **2D** and **E**), among which si-289 had the most significant interference effect, with the mRNA level silenced by about 70% and the protein level reduced by about 50%. Therefore, si-289 was selected for subsequent experiments.

### 3.2 The CCK-8 Assay was Utilized to Assess the Impact of Various Concentrations of FMN on Cell Vitality

To achieve the desired therapeutic effect while minimizing drug side effects, experiments were conducted to determine the optimal concentration of *FMN*. Different concentration gradients of *FMN* were prepared and applied to macrophages. After 48 hours, the viability of macrophages was measured using the CCK8 assay. When macrophages were treated with *FMN* at concentrations of 2, 5, 7.5, and 10 μM for 48 hours, cell viability decreased significantly (P < 0.05) (Fig. **3**). Nevertheless, despite this decrease, cell viability remained at approximately 80-90%, a level suggesting that cells still retained their fundamental metabolic and physiological functions. Moreover, previous research has demonstrated that 10 μM of *FMN* can elicit a potent cellular response [28, 29]. Therefore, this concentration was chosen for the subsequent experiments in this study.

### 3.3 The Effects of FMN and α7nAChR on Lipid Phagocytosis of M1/M2 Cells were Detected by Oil Red O Staining

Macrophages may take up and accumulate lipids and form lipid droplets under specific physiological or pathological conditions [8]. To investigate the effect of *FMN*-mediated α7nAChR on lipid uptake and accumulation by macrophages, lipid content in M1 and M2 macrophages was examined by Oil Red O assay. Compared with the M1+si-NC+*FMN* group, the amount of intracellular lipid phagocytosis was increased in the M1+si-α7nAChR+*FMN* group (Fig. **4A**). Compared with the blank M2 cells, the phagocytic lipid content in the M2+si-NC+*FMN* group was reduced. Compared with the M2+si-NC+*FMN* group, the intracellular lipid phagocytosis was increased in the M2+si-α7nAChR+*FMN* group (Fig. **4B**). These results suggest that *FMN* can reduce lipid deposition in macrophages by improving inflammatory response through immunomodulatory or tissue repair functions, and this regulatory function requires the mediation of α7nAChR.

### 3.4 The mRNA Expression of M1/M2 Cells in Each Group was Detected by RT-qPCR

M1 and M2 macrophages exhibit distinct functional roles in disease progression. The analysis of their associated inflammatory markers serves critical functions in clinical diagnostics, therapeutic monitoring, prognostic assessment, mechanistic investigation, and personalized treatment strategies [4, 5]. To elucidate the regulatory effects of *FMN* mediated through α7nAChR on macrophage polarization during atherosclerotic plaque development, we performed RT-qPCR analysis of polarization-specific markers: M1-associated markers (COX-2, IL-1β, IL-6) and M2-associated markers (SHP1, HO-1). The expression of HO-1, SHIP1, COX-2, IL-6, and IL-1β was increased in the M1+si-NC+*FMN* group cells compared with blank M1 cells. Compared with the M1+si-nc+*FMN* group, the mRNA expression of SHIP1, COX-2, IL-6, and IL-1 in the M1+si-α7nAChR+*FMN* group decreased (Fig. **5A**-**E**). The expression of COX-2, IL-6, and IL-1β in the M2+si-NC+*FMN* group was significantly lower than that in blank M2 cells, while the expression of HO-1 and SHIP1 was significantly higher. Compared with the M2+si-nc+*FMN* group, the expressions of COX-2, IL-6, and IL-1β in the M2+si-α7nAChR+*FMN* group were significantly increased, and the expressions of HO-1 and SHIP1 were significantly decreased (Fig. **5F**-**J**). These results suggest that *FMN* can regulate the polarization of macrophages through α7nAChR; that is, *FMN* inhibits the polarization of macrophages to M1 type and promotes the polarization of macrophages to M2 type, thereby reducing inflammation and inhibiting the progression of atherosclerotic plaque, which may provide a new strategy for the treatment of atherosclerosis.

### 3.5 The Protein Expression of M1/M2 Cells in Each Group was Detected by Western Blot

In order to define the *FMN* role in macrophage polarization effect, we use the immune protein imprinting to detect the protein expression level of the M1/M2-related markers. Compared with blank M1 cells, the protein expressions of iNOS, CD68, p-JAK2, and p-STAT3 in the M1+si-NC+*FMN* group were significantly decreased, and the protein expressions of CD206 and α7nAChR were significantly increased. Compared with the M1+si-NC+*FMN* group, the protein expressions of iNOS, p-JAK2, and p-STAT3 in the M1+si-α7nAChR+*FMN* group were significantly increased, the protein expressions of CD206 and α7nAChR were significantly decreased, and there was no significant difference in the expression of CD68 (Fig. **6A**-**G**). Compared with blank M2 cells, the protein expressions of iNOS, CD68, p-JAK2, and p-STAT3 in the M2+si-NC+*FMN* group were significantly decreased, and the protein expressions of CD206 and α7nAChR were significantly increased. Compared with the M2+si-NC+*FMN* group, the protein expressions of iNOS, CD68, p-JAK2, and p-STAT3 in the M2+si-α7nAChR+*FMN* group were significantly increased, and the protein expressions of CD206 and α7nAChR were significantly decreased (Fig. **6H**-**N**). These results suggest that *FMN* can reduce inflammation and promote tissue repair and remodeling by mediating α7nAChR to reduce the inflammatory response of M1 macrophages and promote polarization to M2 macrophages. *FMN*-mediated α7nAChR plays a positive role in reducing the formation of atherosclerotic plaques in porridge.

## 4 DISCUSSION

As a chronic inflammatory disorder, atherosclerosis pathogenesis encompasses endothelial dysfunction, foam cell formation, and a persistent inflammatory response [30]. This research uncovers a novel mechanism: *FMN* regulates macrophage polarization and lipid metabolism *via* the α7nAChR/JAK2/STAT3 signaling axis, thereby presenting a potential therapeutic target for atherosclerosis. This discovery not only broadens our understanding of the role of cholinergic anti-inflammatory pathways in cardiovascular diseases but also furnishes an experimental foundation for formulating anti-atherosclerosis strategies centered around natural compounds.

The regulation of lipid metabolism by *FMN* demonstrates significant effects on macrophage polarization, with observed reductions in lipid deposition in M1 macrophages and enhanced lipid clearance in M2 macrophages. These beneficial effects were abolished upon α7nAChR deletion, aligning with the findings of Yang A *et al*., who reported that α7nAChR agonists could inhibit foam cell formation *via* cholinergic anti-inflammatory pathways [31]. Notably, our results strongly support the established concept that macrophage phenotype switching critically influences plaque stability [11], suggesting that *FMN* may promote plaque stabilization by facilitating the transition of plaque-associated macrophages toward the M2 phenotype. Recent structural studies on α7nAChR have provided additional mechanistic insights, revealing that allosteric modulation can significantly influence receptor functionality [32]. These structural biology findings offer compelling support for *FMN*' mechanism of action. While *FMN* appears to exhibit broader immunomodulatory properties compared to synthetic α7nAChR agonists, its specificity requires further investigation. At the level of inflammation regulation, we found that *FMN* significantly down-regulated the pro-inflammatory factors of M1 macrophages, such as COX-2, IL-1β, and IL-6, and up-regulated the anti-inflammatory factors of M2 macrophages, such as HO-1 and SHIP1. This result is consistent with the work of Wu X's team on α7nAChR-mediated immune regulation; that is, this receptor plays a central role in anti-inflammatory responses [33]. Notably, the induction of HO-1 by *FMN* suggests that it may activate the Nrf2 antioxidant pathway [34], which is similar to the anti-inflammatory mechanism of natural compounds, such as resveratrol [35]. This provides a new perspective for understanding the pleiotropic anti-inflammatory effects of *FMN*.

The JAK/STAT pathway serves as a crucial intracellular signaling mechanism that governs diverse cellular functions and gene expression, playing a pivotal role in modulating inflammation and immune responses [36, 37]. Extensive research has demonstrated the involvement of JAK/STAT signaling in the inflammatory regulation of atherosclerosis therapy [38-40]. Specifically, the JAK2/STAT pathway enhances and sustains the pro-inflammatory characteristics of macrophages, resulting in elevated secretion of inflammatory mediators such as IL-6 and vascular cell adhesion molecule-1 (VCAM-1), thereby exacerbating atherosclerotic progression [12, 13]. *FMN*, a class of natural compounds with potent anti-tumor and anti-inflammatory properties [41-42], exerts its effects in part through modulation of the JAK2/STAT3 signaling pathway. Previous studies have reported that *FMN* suppresses endometriosis progression by regulating p27, pSTAT3, and progesterone receptors [43]. Additionally, *FMN* has been shown to mitigate inflammation in cerebral ischemia-reperfusion injury in rats by targeting the JAK2/STAT3 pathway [25]. In cervical cancer, *FMN* inhibits tumor progression by downregulating MYC and STAT3, thereby interfering with PD-L1 activation [44]. These findings collectively suggest that *FMN* possesses significant immunomodulatory and anti-inflammatory potential, positioning it as a valuable natural therapeutic agent. However, the precise molecular mechanisms underlying *FMN*'s regulation of macrophage inflammatory polarization in atherosclerosis remain insufficiently explored, warranting further investigation.

The activation of α7nAChRs triggers a cascade of anti-inflammatory responses that modulate multiple intracellular signaling pathways in monocytes and macrophages [45-47]. In macrophages, α7nAChR-mediated activation of the JAK2/STAT3 signaling pathway suppresses inflammatory cytokine production [25, 44]. This process involves α7nAChR-induced JAK2 aggregation and phosphorylation, which subsequently phosphorylates STAT3 (p-STAT3). The resulting p-STAT3 dimers then translocate to the nucleus, where they exert negative regulatory effects on inflammatory cytokine expression [44]. As a critical node of *FMN*'s mechanism of action, the JAK2/STAT3 pathway holds substantial theoretical importance in understanding its regulatory effects. Our findings not only support Luo P *et al*.'s observation that STAT3 contributes to atherosclerotic inflammation [48], but also reveal a novel "rebound" phenomenon of STAT3 phosphorylation following α7nAChR deficiency, providing fresh insights into receptor-pathway interactions. Nevertheless, given the JAK/STAT pathway's involvement in numerous physiological processes, comprehensive evaluation of *FMN*'s long-term safety profile remains necessary.

The pathogenesis of atherosclerosis involves intricate interactions among multiple inflammatory signaling pathways, including the NF-κB pathway, TLR signaling, and oxidative stress responses [49]. Activation of α7nAChR exerts anti-inflammatory effects by suppressing IκB phosphorylation, thereby preventing NF-κB pathway activation and subsequent transcription of proinflammatory cytokines [47, 50]. This mechanism is exemplified by the α7nAChR agonist PNU282987, which attenuates LPS-induced inflammatory damage through inhibition of TLR-4/MyD88 signaling and downregulation of NF-κB expression [49]. Emerging evidence positions the JAK2/STAT3 pathway as a crucial link between α7nAChR signaling and NF-κB regulation, with demonstrated significance across various disease states [51]. Notably, STAT3 phosphorylation can functionally substitute for α7nAChR activation to suppress NF-κB activity [52]. While numerous studies have documented *FMN*'s capacity to inhibit NF-κB activation [53-55], the precise interplay between *FMN*, α7nAChR, and NF-κB signaling within the context of atherosclerotic progression requires further elucidation. These knowledge gaps highlight the need for deeper investigation into *FMN*'s mechanistic role in atherosclerosis-related inflammatory pathways.

This study represents the first comprehensive demonstration of *FMN*'s mechanism in regulating macrophage polarization through the α7nAChR/JAK2/STAT3 axis, offering novel experimental support for the cholinergic anti-inflammatory pathway's role in cardiovascular diseases. These findings align with our prior *in vivo* investigations [27], where ApoE-/- mice treated with *FMN* at doses of 15, 30, and 60 mg/kg exhibited dose-dependent improvements in key atherosclerotic parameters, including aortic plaque area, fibrous cap integrity, and lipid deposition. The treatment concurrently reduced serum levels of atherogenic lipids (LDL-C, ox-LDL, TC, and TG) while modulating inflammatory markers through α7nAChR upregulation and suppression of JAK2/STAT3 phosphorylation. These collective results robustly demonstrate *FMN*'s anti-atherosclerotic effects through JAK/STAT pathway inhibition and α7nAChR activation.

## STUDY LIMITATIONS

While these findings are significant, certain limitations warrant consideration. The use of THP-1 cell lines, which exhibit distinct lipid metabolism characteristics compared to primary macrophages, may affect the broader applicability of these conclusions. Studies on the correlation between the effects of *FMN* and dose still need to be strengthened further. Furthermore, despite *FMN*'s demonstrated pharmacological versatility, its clinical translation faces challenges due to poor bioavailability and solubility [56]. This limits clinical translational potential and requires further elucidation of off-target or overlapping pathways affecting anti-inflammatory effects. Nevertheless, the concordance between our animal studies and cellular data strengthens the validity of our conclusions. From a translational medicine perspective, available safety data support the development potential of *FMN* as a long-term preventive agent and as an anti-atherosclerotic lead compound. However, there is still a lack of data from systematic studies on the acute or chronic toxicity of *FMN* in humans. Therefore, future studies should focus on optimizing the drug delivery system of *FMN* to improve its bioavailability, verify its efficacy and safety in more clinically relevant animal models, and explore the potential synergistic effect of *FMN* combined with statins, so as to provide a more adequate scientific basis for clinical application.

## CONCLUSION

In conclusion, this study demonstrates that *Formononetin* exerts therapeutic effects against atherosclerosis by modulating macrophage polarization and inflammatory responses through the α7nAChR/JAK2/STAT3 signaling axis. The activation of the cholinergic anti-inflammatory pathway promotes M2 macrophage polarization, while suppression of the JAK2/STAT3 pathway attenuates inflammation. These findings not only enhance our understanding of inflammatory regulation in atherosclerosis but also bridge a knowledge gap regarding the role of α7nAChR in metabolic inflammation. Furthermore, this work establishes a theoretical foundation and identifies potential therapeutic targets for anti-atherosclerotic drug development. Future investigations will focus on validating *FMN*'s efficacy in animal models, optimizing dosage regimens and administration methods to maximize its anti-atherosclerotic effects. We will also conduct comprehensive preclinical assessments, including safety profiling and pharmacokinetic studies, to facilitate clinical translation. Additionally, we plan to employ multi-omics approaches to elucidate novel mechanistic pathways of *FMN*, thereby accelerating its transition from basic research to clinical applications.

## Figures and Tables

**Fig. (1):**
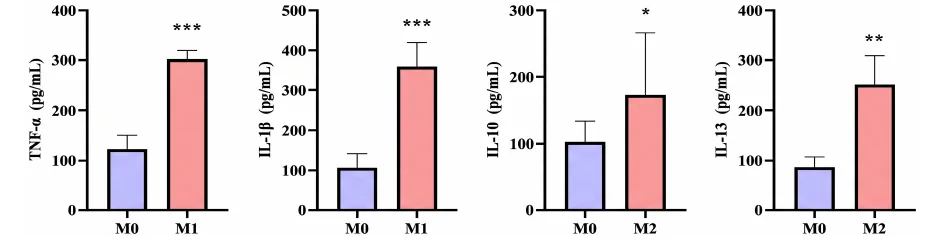
ELISA detected the levels of inflammatory or anti-inflammatory factors, including TNF-α, IL-1β, IL-10, and IL-13, in the cell supernatant. *: *P* < 0.05, **: *P* < 0.01, ***: *P* < 0.001.

**Fig. (2):**
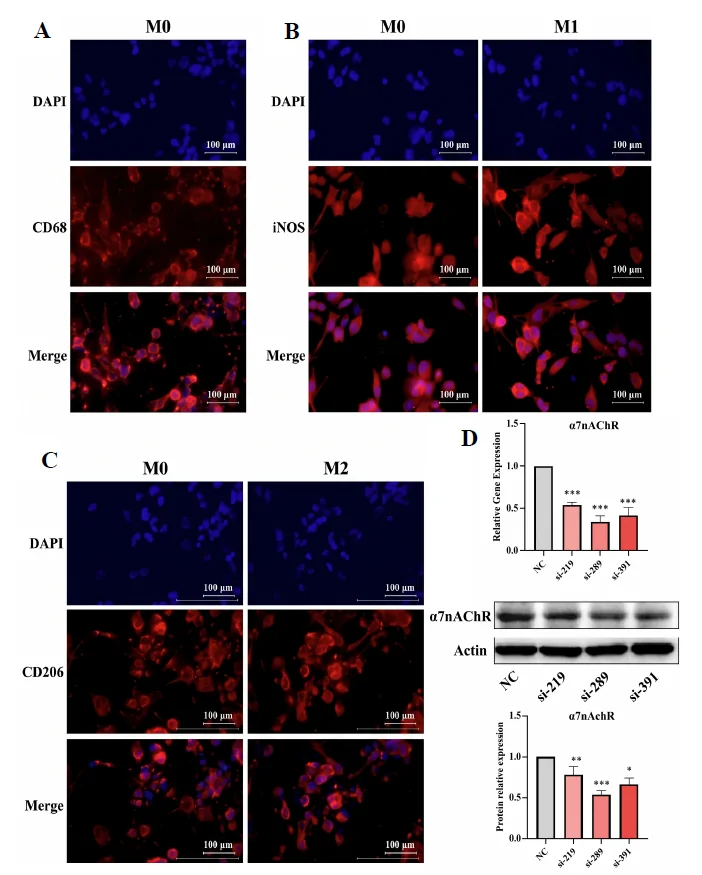
Macrophages were identified by immunofluorescence, and the silencing effect of α7nAChR was detected by RT-qPCR and Western blot. (**A**) THP-1 cells were stimulated with 320 nmol/L PMA for 30 h to induce M0 macrophages. (**B**) M0 macrophages were stimulated with 20 ng/mL IFN-γ and 100 ng/mL LPS for 48 h to induce M1 macrophages. (**C**) M0 macrophages were stimulated with 20 ng/mL IL-4 and IL-13 for 48 h to induce M2 macrophages. The image scale was 100 μm. (**D**) The expression of α7nAChR mRNA in macrophages was detected by RT-qPCR. (**E**) The expression of α7nAChR protein in macrophages was detected by Western blot. *: *P* < 0.0, **: *P* < 0.01, ***: *P* < 0.001.

**Fig. (3):**
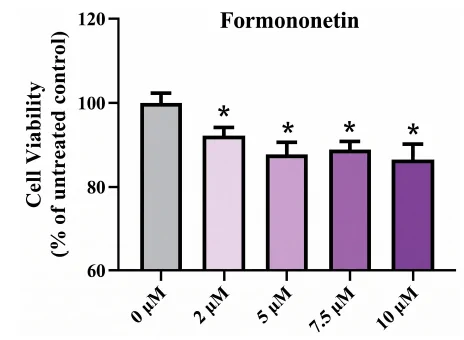
Effects of different concentrations of *FMN* on cell proliferation detected by CCK8. * indicates *P* < 0.05 compared with 0 μM group.

**Fig. (4):**
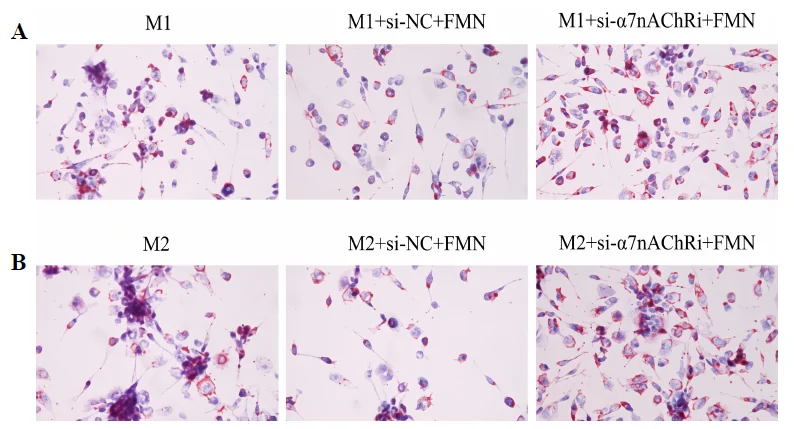
The effect of *FMN* on lipid phagocytosis of M1 and M2 cells was observed by oil red O staining. (**A**) *FMN* treatment significantly reduced lipid accumulation in M1 macrophages (decreased red staining area) compared with the M1 group, while the inhibitory effect of *FMN* was partially reversed after α7nAChR knockdown. (**B**) *FMN* treatment enhanced the lipid clearance capacity of M2 macrophages (decreased red-stained areas) compared with the M2 group, whereas downregulation of α7nAChR attenuated this effect. Scale bar: 100 μm.

**Fig. (5):**
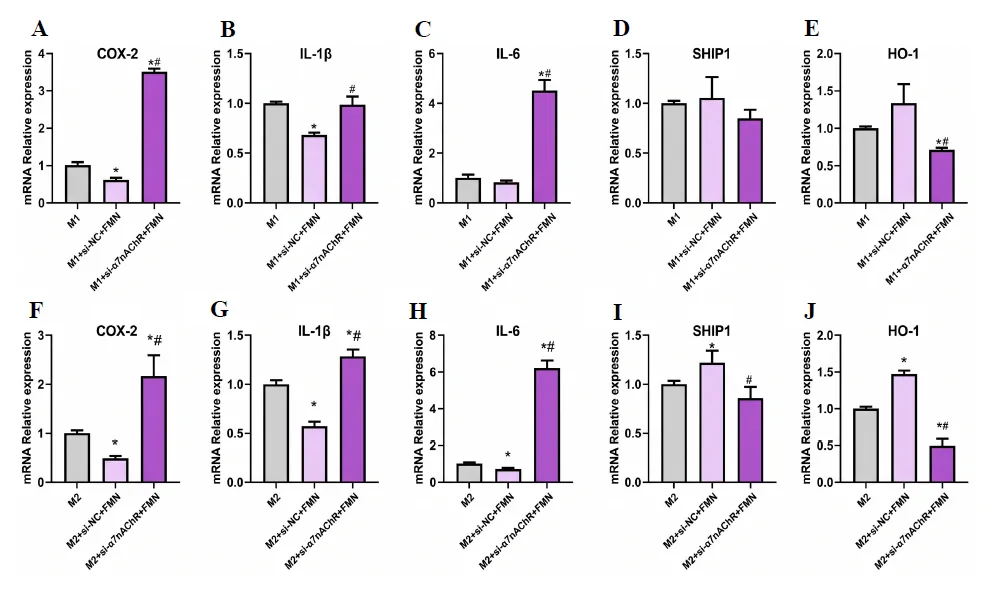
RT-qPCR was used to detect the mRNA expression levels of inflammatory factors such as COX-2, IL-1β, IL-6, SHIP1, and HO-1 in M1 and M2 cells of each group. (**A-E**) M1 macrophages. (**F-J**) M2 macrophages. * indicates comparison with M1/M2 group, *P* < 0.05; # indicates comparison with M1/M2+si-NC+*FMN* group, *P* < 0.05.

**Fig. (6):**
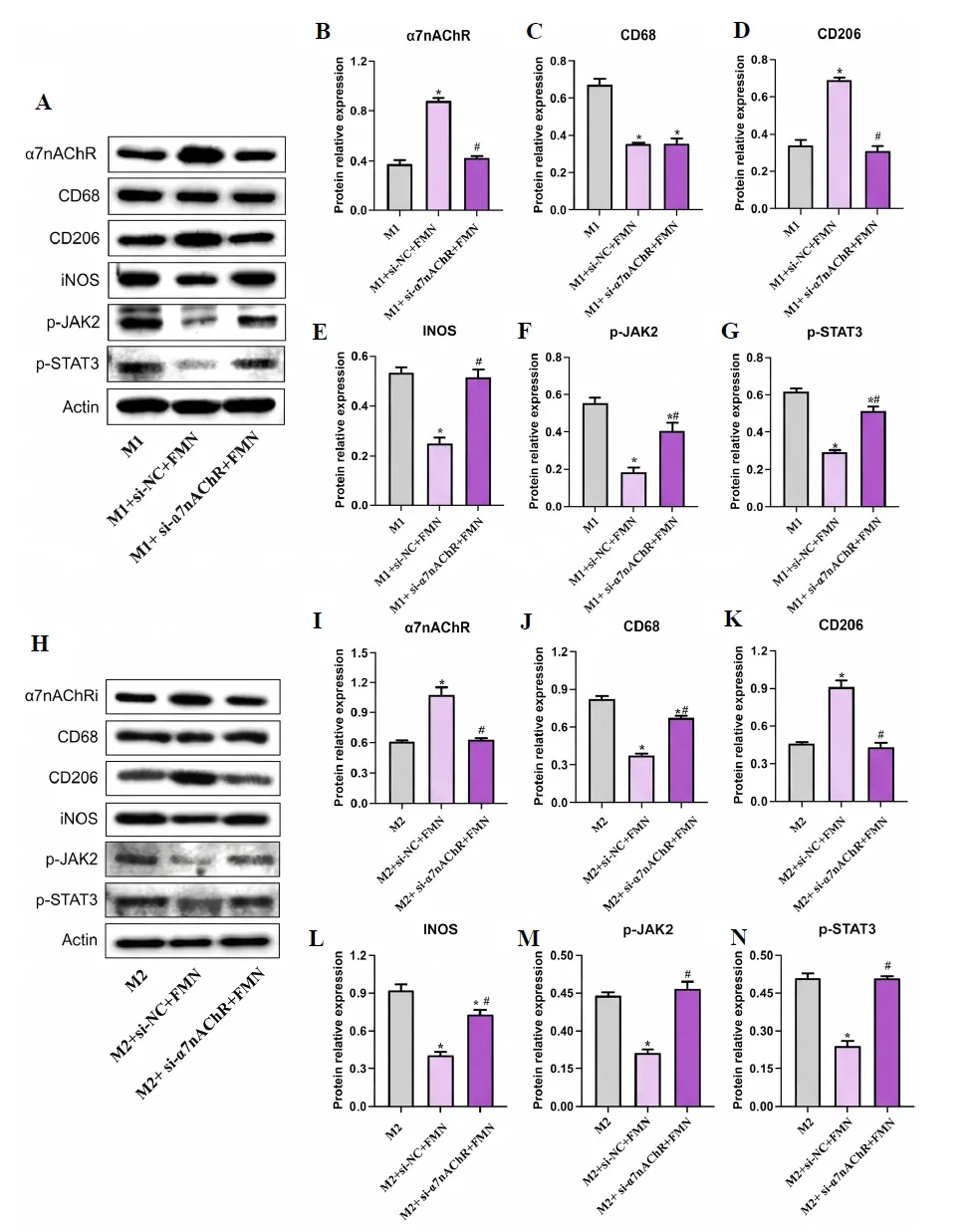
The protein expression of α7nAchR, CD68, iNOS, p-JAK2, and p-STAT in M1/M2 cells of each group was detected by Western blot. (**A-G**) M1 macrophages. (**H-N**) M2 macrophages. * indicates *P* < 0.05 compared with M1/M2 group; # indicates *P* < 0.05 compared with M1/M2+si-NC+*FMN* group.

**Table 1:** Primer sequences for QPCR.

**Primer Name**	**Primer Sequence**
*COX-2*	Forward 5’-CAAAACCGAGGTGTATGTATGA-3’
	Reverse 5’-CCCTTGAAGTGGGTAAGTATGTAG-3’
*IL-1β*	Forward 5’-GCACGATGCACCTGTACGAT-3’
	Reverse 5’-TGGAGAACACCACTTGTTGC-3’
*IL-6*	Forward 5’-CTGGTCTTTTGGAGTTTGAGG-3’
	Reverse 5’-TGGCATTTGTGGTTGGGT-3’
*HO-1*	Forward 5’-TCCGCAACCCGACAGCA-3’
	Reverse 5’-GAAGTAGACAGGGGCGAAGA-3’
*SHIPl*	Forward 5’-AGCGACGAATCCTATGGCG-3’
	Reverse 5’-CTGGATTCATCACGCTCCGT-3’
*α7nAChR*	Forward 5’-GGAAGCTTTACAAGGAGCTG-3’
	Reverse 5’-TTTCCAAATCTGGCCATCTGG-3’
*β-actin*	Forward 5’-TGGCACCCAGCACAATGAA-3’
	Reverse 5’-CTAAGTCATAGTCCGCCTAGAA-3’

## Data Availability

The data that support the findings of this study are available on request from the corresponding author (QH), upon reasonable request.

## LIST OF ABBREVIATIONS

Arg-1: Arginase-1
AS: Atherosclerosis
FMN: Formononetin
IFN: Interferon
IL-1β: Interleukin 1 Beta
IL-6: Interleukin 6
LPS: Lipopolysaccharide
MMPs: Matrix Metalloproteinases
P-STAT3: Phosphorylates STAT3
TNF-α: Tumor Necrosis Factor-Alpha
